# Human and environmental gradients predict catch, effort, and species composition in a large Micronesian coral-reef fishery

**DOI:** 10.1371/journal.pone.0198068

**Published:** 2018-05-31

**Authors:** Javier Cuetos-Bueno, Dalia Hernandez-Ortiz, Curtis Graham, Peter Houk

**Affiliations:** 1 University of Guam Marine Laboratory, Mangilao, Guam; 2 Chuuk Department of Marine Resources, Weno, Chuuk, Federated States of Micronesia; Department of Agriculture and Water Resources, AUSTRALIA

## Abstract

The consistent supply of fresh fish to commercial markets may mask growing fishing footprints and localized depletions, as fishing expands to deeper/further reefs, smaller fish, and more resilient species. To test this hypothesis, species-based records and fisher interviews were gathered over one year within a large, demand-driven coral-reef fishery in Chuuk, Micronesia. We first assessed catch statistics with respect to high windspeeds and moon phases that are known to constrain both catch and effort. While lower daily catch success was predicted by higher windspeeds and greater lunar illumination, total daily landings fluctuated less than fishing success across environmental gradients. Instead, daily landings were mainly driven by the number of flights from Chuuk to Guam (i.e., international demand). Given that demand masked local drivers of overall catch volume, we further evaluated species-based indicators of fisheries exploitation. Most target species (75%) had either a positively skewed size distribution or proportional contributions that were dependent upon favorable conditions (i.e. season and moon phases). Skewed size distributions indicated truncated growth associated with fishing mortality, and in turn, suggested that size-based management policies may be most effective for these species. In contrast, environmentally-constrained catch success indicated species that may be more susceptible to growing fishing footprints and may respond better to gear/quota/area policies compared to size policies. Species-based responses offered a simplified means to combine species into fisheries management units. Finally, a comparison of commercial and subsistence landings showed higher vulnerability to fishing among species preferentially targeted by commercial fisheries, offering new insights into how commercial harvesting can disproportionately impact resources, despite having lower annual catch volumes.

## Introduction

Coral-reef fisheries provide food security, economic prosperity, and recreation for island nations [1,2]. Yet, unsustainable exploitation driven by growing human populations, commercialization of fisheries, technological advances, and globalization of markets threatens the sustainability of reef fisheries worldwide [3]. While commercial landings are typically less than 50% of subsistence landings for most island nations [4], they may have disproportional impacts on both the fishery and the ecosystem. Disproportional impacts from commercial landings can arise because profit-driven harvesting often focuses on the largest species that have slow growth rates and high ecological functionality [5–7]. Once localized depletions occur, expansions of commercial fishing effort typically follow because investments in fishing infrastructure are engaged [8–10]. Indeed, many studies have documented that distance to commercial markets is a strong predictor of both stock biomass and ecosystem condition across differing geographical scales [9–12].

While fisheries depletions have been reported over the last six decades [13,14], many commercial coral-reef fisheries have maintained consistent, demand-driven landings [15,16]. Consistent revenues and landings despite localized depletions are dangerous because they provide a false sense of sustainable harvesting to fishers and consumers that purchase fish in local and international markets. This phenomenon where local ecosystem signals are diluted or masked by seafood markets is not exclusive to coral-reef fisheries, and has been described for other fisheries (e.g. North Sea cod fishery) [17]. Several factors can contribute to the perception of sustainable fisheries in the face of depletion, including: i) changes in the catch composition as preferred species and sizes are replaced by less-desirable but more productive species or size classes [18], ii) increasing fishing effort to compensate for reductions in fishing success [19], iii) local expansion of fishing activities to further and deeper grounds [20], and iv) trade from other locales following local economic extinctions [21,22]. Combined, these factors can maintain or even increase the supply of fresh fish being provided to markets while masking the effects of localized depletions.

For over a decade, ~150 metric tons yr^-1^ of reef fish from Chuuk, Federated States of Micronesia, have been supplied on average to Guam [23]. Demand on Guam was linked with continued human population growth, limited reef habitat, and the depletion of coastal fisheries [18,24,25]. Chuuk exports became increasingly driven by demand over this time period, with governmental food allowances in Guam explaining a growing proportion of the variance in daily export records within each month from Chuuk. Cuetos-Bueno and Houk [23] provided further details regarding the study of Chuuk-Guam reef-fish trade, but their findings hinted towards healthy and sustainable stocks in Chuuk given the consistent supply of fresh fish to Guam markets. However, fisheries-independent surveys and anecdotal evidence began reporting ecosystem consequences of Chuuk’s expanding fishing footprint [12,26–28]. Evidence included declining fish biomass and corals and calcifying substrate cover that were linked with distances from population centers, and fishers reporting reductions in fish sizes, abundances, and fishing success. The contrasting views provided by fish export records and fisheries-independent surveys were reconciled in the present study of commercial reef fish landings in Chuuk.

The present study provided a deeper examination into species-based catch records for one of the largest coral-reef fisheries in Micronesia. Detailed commercial catch records and coupled fisher interviews were collected over one year to (i) investigate drivers of catch-and-effort statistics and (ii) assess populations of target species that comprised 75% of landings. Catch and effort were first examined across geographic sectors and seasonal environmental gradients because both are related to fishing access and commercial demand. The magnitude of these relationships provided an initial indication of fishery status that was tested with subsequent species-based evaluations. For example, fishing success was expected to increase with favorable weather conditions that dictate fishing access. If this is true, then understanding which species were most sensitive to environmental gradients becomes desirable from a management perspective. Family and species-based examinations were conducted to depict stock status. These included comparing fish sizes to their proportional contribution to landings within each dominant fish family. The premise was that smaller species within each family and more productive species lower in trophic position, may be taking the place of larger vulnerable species, especially predators. Next, target species comprising 75% of total landings from all trophic levels were further examined to determine if catch success was dependent on favorable environmental regimes, and whether skewed size-frequency distributions existed. Both are potential indicators of impacted stocks and are linked with differing management strategies [29,30]. Finally, we integrated data from a previous study to compare commercial versus subsistence fishery landings and contrast the roles of subsistence versus commercial exploitation [27].

## Materials and methods

### Study area

**Fig 1:**
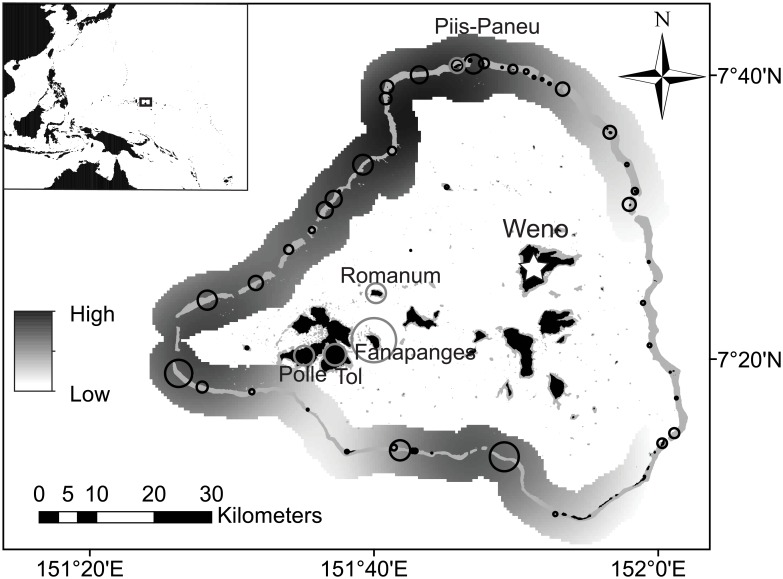
Map of Chuuk lagoon, including the capital island of Weno (white star), where fish markets and exporters are located. Map also depicts 1) contribution of specific reef-sections to overall recorded commercial landings (black circles, proportional from 0.1% to 12.5%), 2) estimated distribution of landings across the reef barrier (gray gradient), and 3) proportional contribution to overall ladings of top five fishing communities (gray circles, proportional from 6% to 52%).

Chuuk lagoon, Federated States of Micronesia, is one of the largest lagoons in the world, with over 2,000 km^2^ of reef and lagoon habitat, and 200 km of barrier reef (Fig 1). With a population of 36,152 [31], the amount of coral-reef habitat per-capita is among the largest in Micronesia. Marine resources have sustained Chuuk’s societies for generations, while being traditionally managed by individual reef owners. Yet, traditional reef ownership and management has weakened over the last decades. Alongside, commercial resource exploitation has been increasing due to (i) a lack of alternative sources of income for the growing population, (ii) easy access to both local and international markets, (iii) a large governmental distribution of fiberglass motorized boats in the 1980s and 1990s, and iv) limited regulation and enforcement over the last decades. As a result, subsistence fishers in Chuuk lagoon are increasingly turning to commercial fishing to provide a steady income [23,27,32]. Chuuk’s reefs are still in good condition when compared across the populated islands of Micronesia [12]. Yet, the growing fishing footprint is apparent, as reefs near inhabited islands have some of the lowest fish abundances reported in Micronesia, while sites along the barrier reef have some of the highest [12,28]. Thus, there is evidence for a growing spatial footprint in Chuuk’s fisheries to meet social needs and market demands.

### Data collection

#### Fisheries-dependent data

Comprehensive fisheries-dependent datasets were collected through a combination of commercial fisher interviews and catch monitoring between November 2013 and October 2014. Daily visits to the thirteen prominent fish markets were conducted to collect fisher interviews (n = 839 interviews). Interviews gathered information regarding fishing location, gear, expenses, and other relevant fishing and socioeconomic factors. Three of the largest markets were selected to generate data on catch composition using a photographic monitoring system. During each visit, all fish being sold in the market were photographed on top of a measuring board (n = 79,097 individual fish). Individual photographs were entered into a dedicated database where fish were identified, and length was measured to the nearest cm fork length. In addition to lengths, a subset of weights of individual fish were also collected to create local estimates of length-weight relationships for commonly caught species [33]. Fish lengths were then converted to biomass using local length-weight parameters when possible [34], or regional parameters from other monitoring programs in Micronesia when not available for Chuuk. To compliment the photographic surveys, visual estimates of catch composition were opportunistically collected in other markets. During these surveys, individual fish from landings were identified to the highest taxonomic resolution possible, and sizes were visually estimated to the nearest cm fork length (n = 37,894 individual fish). Lastly, the total daily landings of reef fish purchased by each market were recorded directly from fish market purchase records.

#### Environmental data

Windspeed data were collected from the weather station at the Weno Airport (Chuuk) (http://www7.ncdc.noaa.gov/CDO). Windspeeds were binned into 1 km/h groups. Lunar data were collected from the United States Naval Observatory webpage (http://aa.usno.navy.mil). Lunar days were defined by applying a value of 0 to full moon days, and counting the days away from the full moon for other moon-calendar days (i.e., both directions from the full moon). Moon calendar days were also binned into three moon-phase categories, using equal ranges of moon illumination (new-moon = 0–33% illumination, half-moon = 34–66% illumination, and full-moon = 67–100% illumination). Lastly, four different inter-annual seasons were defined as spring (March to May), summer (June to August), autumn (September to November), and winter (December to February).

#### Ethics statement

No institutional review board existed in Chuuk State, Federated States of Micronesia at the time of the study, and no special approval was required by our funding sources. However, monitoring protocols were developed in accordance with principles expressed in the Declaration of Helsinki, and were approved by the Chuuk Government. Fisher interviews started by explaining the objective of the study and the use of the information they provided. Further, it was explained that their responses would remain confidential, and that they will not be identified in any report or publication. Written consent was not obtained due to prevalent illiteracy among Chuuk fishers, and sensitivity regarding signing forms containing their names. Instead, verbal consent to participate in the survey was implicitly understood by the fact that fishers participated in the interviews since those who did not provide consent were not interviewed.

### Data preparation and analysis

Total annual landings were estimated for each market by multiplying the following estimates: (i) the mean daily landings, (ii) the mean weekly frequency of fish landings, and (iii) the number of actual operational weeks within the year. Total commercial landings were then estimated by combining data from each market and adding commercial landings that were directly sold to fish exporters. A previous study examined export records from the Chuuk Food Safety Office to corroborate export estimates used in the present study [23]. Economic value of total landings was estimated using 2015 retail reef fish prices of US$3.85 per kilogram [23].

The percent contribution of differing fishing methods to overall landings was calculated based on responses to interview questions and the overall landings for each associated fishing trip (n = 839 fishing trips). Trips that used more than one fishing method were excluded (n = 41). Fishing location maps were then created by linking catch records with interviews where fishing location was recorded (n = 698 fishing trips). Fishing location maps served to highlight the origin of landings. Location maps were smoothed by applying a kernel density function built into the ArcGIS platform [35], depicting the relative contribution of each reef section to overall landings.

Next, relationships between environmental drivers and fishing success were evaluated, as strong or growing dependences of fishing success upon environmental gradients/cycles may be linked with compromised stock status [5,36]. Polynomial regression models were used to understand whether mean daily fisher landings were dependent upon daily environmental factors (i.e., lunar days and windspeeds). Linear and polynomial models were evaluated based on their fit (R^2^), confidence interval (P-values), and complexity (Akaike information criterion scores, AIC). Best fit models were tested for normality of residuals. Seasonal trends in landings were also examined by aggregating data by month within each season, and identifying any months that were significantly higher or lower than the annual mean (± 1 SD). Total landings were also compared across week days using Kolmogorov—Smirnov tests.

We next focused on the dominant components of the fishery by selecting fish families that represented at least 5% of overall landings (n = 6 families), and species within these families that represented at least 1% of family-level biomass (n = 49 species). Together, these 49 species accounted for 83% of the total annual landings. The relationships between the proportional contribution to family landings and species-based maximum lengths (L_max_) were then evaluated within each family. The premise for these analyses was that commercial fisheries preferentially target larger fishes/species opportunistically, and over time may shift the composition of the landings towards smaller fishes lower in trophic position [18,30]. In support, larger-bodied species, especially predators, comprise the majority of fish biomass on remote and less fished reefs [37–40]. Further, the expected pattern of biomass accumulation within larger species is also supported by foundational ecological principles governing biomass accumulation within food webs [41]. Regression models were used to examine biomass versus body-size relationships; the null hypothesis was that a linear relationship existed based upon foundational theory and empirical data from the remote systems described above. Due to the reliance upon accurate size estimations for these analyses, only the photo-monitoring subset of catch composition datasets was used (n = 73,544 individual fish).

We then conducted a series of species-based analyses to assess potential indicators of fishery status with respect to spearfishing, the most dominant form of commercial fishing (86% of overall landings). Proportional contributions to landings were contrasted across differing moon phases and seasons using Kolmogorov—Smirnov tests for the top 20 species that made up 75% of landings. Next, fish size distributions were examined. The skewness of fish size distributions were calculated for all species, and these values were binned into three categories based upon natural breaks [42]. The three categories represented species that were (i) dominated by smaller fish (skewness>0.5), (ii) dominated by larger fish (negative skewness), and (iii) neither (skewness 0–0.5). Herein, the term skewed size-frequency distributions intuitively refers to species with positive skewness >0.5 that were dominated by juveniles. Together, species-based tests provided guidance for our discussion on potential management and policy.

Finally, catch composition estimates from the present study were compared against those from a subsistence fishing study conducted in Chuuk lagoon in 2012 that documented landings from 21 subsistence fishing trips (960 individual fish; [27]). Vulnerability indices for each species were extracted from Fishbase [43,44], and vulnerability-frequency distributions were compared across subsistence and commercial landings using Kolmogorov—Smirnov tests. Further, species were binned into functional groups for a more general comparison between subsistence and commercial landings. Functional groups were defined by family and body size (i.e., large-bodied parrotfishes), with size groupings determined by Jenks breaks of asymptotic lengths defined in Houk et al. [45]. Species vulnerability indices were averaged across each functional group, and a linear regression model examined if vulnerability could predict proportional contributions to commercial and subsistence fishing. Functional groups that contributed >2% of commercial landings were used for these comparisons, representing 91% and 88% of overall commercial and subsistence landings, respectively.

## Results

**Fig 2:**
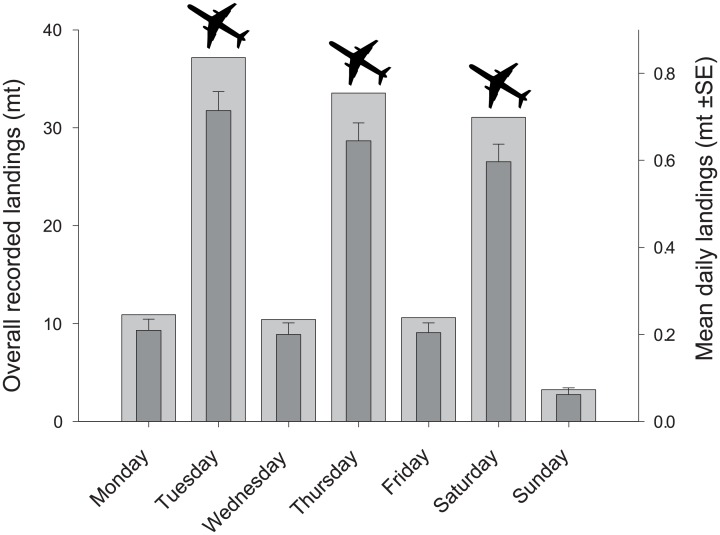
Weekday landings. Mean (dark gray bars) and overall (light gray bars) reef-fish landings for different days of the week with respect to Chuuk-Guam flights (airplane icon, Tuesday, Thursday, and Saturday).

Annual commercial landings in Chuuk were estimated to be 265 mt, with an estimated local value of USD$ 1.02 million. Local commercial sales accounted for 59% of landings, or 157 mt, translating to a mean annual consumption of just 4.3 kg of commercially-caught reef fish per person, suggesting the obvious importance of subsistence fishing in Chuuk. This consumption rate is reflective of high subsistence-to-commercial harvesting ratio in Chuuk (~8:1), which is low compared to other Pacific Island countries (14:1 to 0.7:1 [4]). Exports accounted for the remaining 41%, or 108 mt, which were transported to Guam. Thus, the weekly flights between Chuuk and Guam were a clear driver of daily landings (Fig 2, Table A in S1 Tables; P<0.001, KS test for all comparisons between flight vs non-flight days).

The majority of landings were caught by night-time spearfishing (86%), with secondary contributions from bottom and net fishing, 11% and 3%, respectively. Mean daily catch and income per fisher were estimated at 19.6 kg (±0.3 SE) and $43 (±1 SE) for spearfishing, 23.0 kg (±1.1 SE) and $44 (±3 SE) for bottom fishing, and 12.6 kg (±0.9 SE) and $23 (±2 SE) for net fishing. Spearfishing was the most efficient method. Based on known fishing expenditures captured during interviews, mean fishing costs were estimated at $0.86 kg^-1^ (±0.04 SE) for spearfishing, $1.16 kg^-1^ (±0.11 SE) for bottom fishing, and $1.14 kg^-1^ (±0.24 SE) for net fishing, indicating a 71%, 61%, and 62% profit margin, respectively. Fishing effort focused almost exclusively on the barrier reefs (96% of landings), where fishing success and profitability were highest despite higher travel costs. Accordingly, catch and income were highest for the barrier reef, moderate and variable for patch reefs, and consistently lowest for inner fringing reefs (S1 Fig; Table B in S1 Tables). Along the barrier reef, fishing activity was highest on reefs less exposed to dominant north east trade winds in the western and southern part of the lagoon, and lowest on the windward east side (Fig 1). Yet, overall landings across reef sectors and seasons were tightly coupled with the number of fishing trips (P<0.001, R^2^ = 0.932; for linear relationship; S2 Fig; Table C in S1 Tables), suggesting that factors other than fishing success (i.e. habitat availability and other natural factors that dictate fish abundances) were drivers of the geographical distribution of fishing effort across the barrier reef.

Interestingly, the fishing community associated with Fanapanges, one of the smallest islands within the Chuuk lagoon (672 people, or 1.9% of the population, Fig 1), accounted for 52% of the overall commercial landings. Other important contributions came from the islands of Tol (12.6% landings, and 12.7% population), Polle (11.0% landings, and 4.1% population), Romanum (10.7% landings, and 2.4% population), and Piis-Paneu (6.4% landings, and 1.0% population). Together, fishers from these five islands accounted for 93% of overall commercial landings.

**Fig 3:**
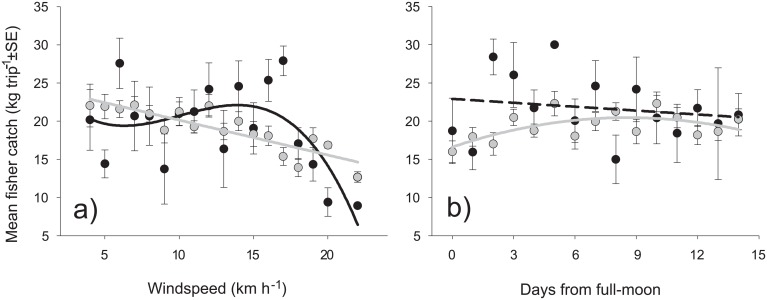
Trends of mean spearfishing (gray dots and lines) and bottomfishing (black dots and lines) catch rates (kg trip^-1^) along a gradient of windspeed (a), and lunar days (b). Decreasing catches were associated with higher windspeeds for both gears, but showed a stronger response for bottomfishing. Spearfishing landings decreased with proximity to full moon, but no significant relationship was found for bottomfishing landings. Solid lines indicate significant fits for polynomial regression models. The dashed line indicates non-significant best-fit polynomial regression.

Diverse results were found when exploring the effects of environmental factors on mean daily and monthly fisher success (kg fisher^-1^ trip^-1^). Significant polynomial relationships existed between spearfishing and daily windspeed (P<0.001, R^2^ = 0.738, y = 24.71–0.46x) and lunar days (P = 0.048, R^2^ = 0.297, y = 16.59 + 0.90x − 0.05x^2^), as lower success was associated with increasing windspeeds and proximity to full moon (Fig 3; Table D in S1 Tables). A similar relationship was observed between bottomfishing success and windspeed (P = 0.037, R^2^ = 0.324, y = 28.10 − 3.25x + 0.37x^2^ − 0.01x^3^), but with a stronger drop of mean landings at high windspeeds. No significant effect of lunar days on bottomfishing success was found.

Monthly catch success fluctuated less compared with daily success. Spearfishing catches remained stable across study months, ranging from a minimum of 15.7±0.7 kg fisher^-1^ trip^-1^ in December to a maximum mean of 22.7±1.3 kg fisher^-1^ trip^-1^ in June (S3 Fig; Table E in S1 Tables). Alternatively, bottomfishing landings were lowest in January (17.9±1.6 kg fisher^-1^ trip^-1^), but peaked in March (34.8±6.5 kg fisher^-1^ trip^-1^). This represented a remarkable 94% increase over two months. While the windy season was often associated with diminished success, the March exception for bottomfishing was notable, and suggested factors beyond weather affected fishing success (e.g., known grouper spawning aggregations).

### Catch composition

**Fig 4:**
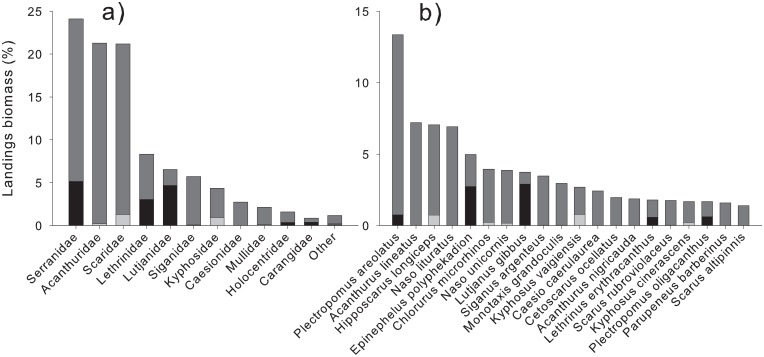
Proportional contributions to overall commercial reef fish landings of main families (a) and top twenty species making 75% of landings (b), caught by spearfishing (dark gray), bottom fishing (black), and net fishing (light gray).

Chuuk’s fishery was characterized by a diverse suite of species (132 recorded), but only a subset of these species within a few families were dominant. Groupers (24% of landings), surgeonfishes (21%), parrotfishes (21%), emperors (8%), snappers (7%), and rabbitfishes (6%) were most frequently caught (Fig 4a; Table F in S1 Tables). The top 20 species accounted for 75% of overall landings (Fig 4b), while just eight species accounted for 50%, and the squaretail coral trout alone, *Plectropomus areolatus*, accounted for 13%. Spearfishing was associated with the greatest diversity in landings, but bottomfishing was most important for snappers (71% of biomass from bottomfishing), trevallies (45%), and emperors (36%), with *Lutjanus gibbus* (78%), *Epinephelus polyphekadion* (55%), *Plectropomus oligacanthus* (38%), and *Lethrinus erythracanthus* (33%) being primary components.

### Species-based assessments

**Fig 5:**
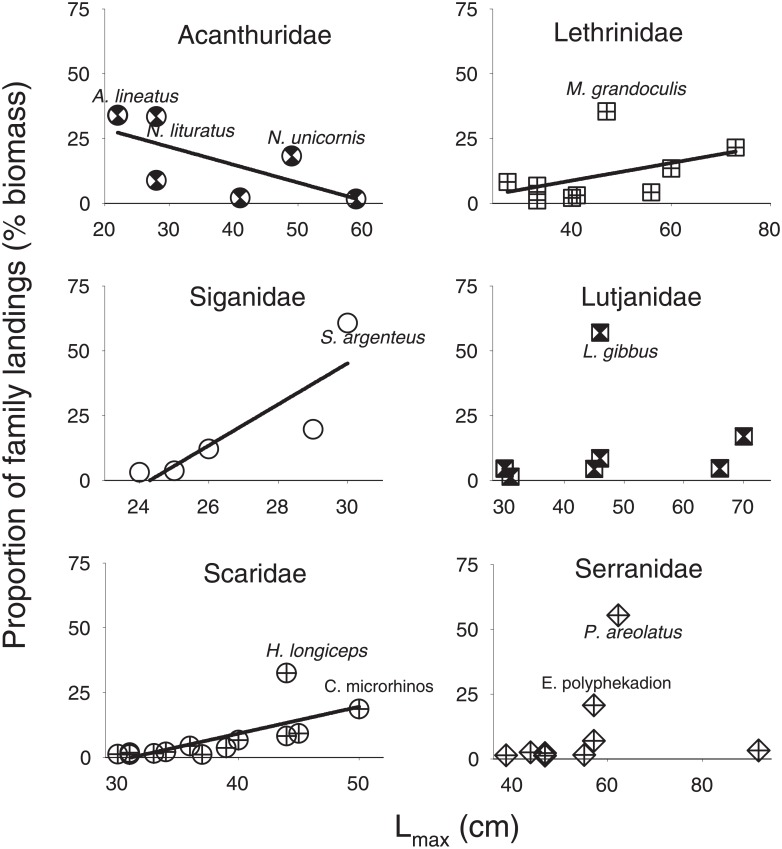
Relationship between species maximum length (L_max_) and proportional contribution to family landings (biomass) for the top six families that dominate Chuuk reef fish commercial landings. Linear relationships were observed for families where larger species comprise most of the landings, with the exceptions of Acanthuridae, where smaller species (*A*. *lineatus* and *N*. *lituratus*) comprised most of the landings. Alternatively, no linear relationships were observed for families dominated by predators (Lutjanidae and Serranidae), where larger species made up a low proportion of landings biomass. Names of species that contributed ≥3% to overall landings are included.

There was a general trend of decreasing proportional contributions from large-bodied species when moving from low to high trophic levels. Biomass within lower trophic-level families was dominated by large-bodied species (i.e. parrotfishes), resulting in a positive linear relationship between maximum lengths and biomass contribution (Fig 5; Table G in S1 Tables). Meanwhile, landings from higher trophic-level families such as groupers and snappers were dominated by medium-sized species (*P*. *areolatus* and *L*. *gibbus*), resulting in no linear relationship between species-based asymptotic lengths and proportional biomass contributions. Finally, one family contrasted these general trends, as surgeonfish landings were dominated by small-sized species (*Acanthurus lineatus* and *Naso lituratus*, preferable foodfish species for local consumption), providing the only negative linear relationship found.

Many of the dominant commercial fishes had positively skewed size distributions and/or were preferentially caught during favorable moon phases and seasons (Table 1; Table H and Table I in S1 Tables). In total, species with skewed distributions or environmentally-constrained catch success made up 57% of the overall landings. Most of these represented larger species within their respective families, and mid-to-high trophic level piscivores and invertivores (large-bodied groupers, emperors, and snappers). In contrast, fishes without catch success being dependent on environmental conditions and without skewed size distributions were mainly a mixture of large- and small-bodied herbivores.

**Table 1:** Twenty species that represented 75% of Chuuk commercial reef-fish landings, their functional group, individual contribution to landings, skewness of size-frequency distribution, and significant (Kolmogorov—Smirnov Test) dependence of fishing success upon environmental conditions (season and moon phase). Species whose fishing success was found to be dependent upon environmental conditions *and* with skewed distribution are highlighted by dark gray background. Species whose fishing success was found to be dependent upon environmental conditions (moon phase and seasonality) *or* with skew distribution highlighted by light gray background.

Species	Functional group	Contribution to overall landings (%)	Skewed size-frequency distribution	Catch success dependent upon environmental conditions
*Plectropomus areolatus*	Large-bodied grouper	13.4	✓	✓
*Naso unicornis*	Large-bodied acanthurid	3.9	✓	✓
*Lutjanus gibbus*	Large-bodied snapper	3.7	✓	✓
*Lethrinus erythracanthus*	Large-bodied emperor	1.8	✓	✓
*Plectropomus oligacanthus*	Large-bodied grouper	1.7	✓	✓
*Epinephelus polyphekadion*	Large-bodied grouper	5.0	✓	
*Kyphosus vaigiensis*	Rudderfish	2.7	✓	
*Kyphosus cinerascens*	Rudderfish	1.7	✓	
*Parupeneus barberinus*	Goatfish	1.6	✓	
*Acanthurus lineatus*	Small-bodied acanthurid	7.2		✓
*Chlorurus microrhinos*	Large-bodied parrotfish	3.9		✓
*Siganus argenteus*	Rabbitfish	3.5		✓
*Monotaxis grandoculis*	Large-bodied emperor	2.9		✓
*Caesio caerulaurea*	Fusilier	2.4		✓
*Cetoscarus ocellatus*	Large-bodied parrotfish	2.0		✓
*Naso lituratus*	Small-bodied acanthurid	7.1		
*Hipposcarus longiceps*	Large-bodied parrotfish	6.9		
*Acanthurus nigricauda*	Small-bodied acanthurid	1.9		
*Scarus rubroviolaceus*	Large-bodied parrotfish	1.7		
*Scarus altipinnis*	Large-bodied parrotfish	1.4		

### Commercial versus subsistence landings

**Fig 6:**
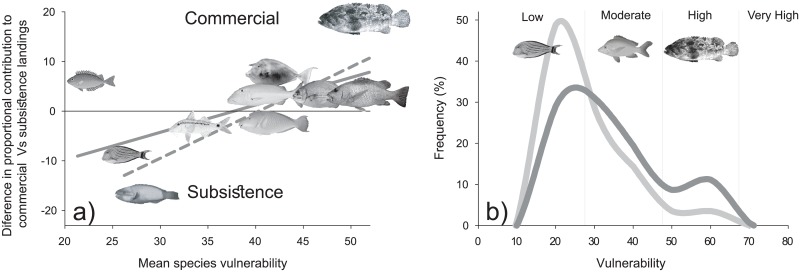
Positive relationship between mean species vulnerability index and difference in proportional contribution to commercial vs subsistence landings for all dominant fish groups (gray solid line), and for all groups but rabbitfishes (gray dashed line, a), and vulnerability frequency distributions within commercial (dark gray line) and subsistence (light gray line) landings [44] (b), revealed increasing vulnerability among commercial landings.

Subsistence landings during the 2012 study were dominated by small-bodied herbivores, mainly parrotfishes and surgeonfishes (Fig 6a). Alternatively, commercial landings reported here were dominated by large-bodied predators, rudderfishes, unicornfishes, and rabbitfishes. Contrasts of catch composition revealed that vulnerability indices were significantly higher for commercial landings than subsistence (P<0.001, KS test; Fig 6b; Table J in S1 Tables). Further, with the exception of rabbitfishes, a positive relationship existed between vulnerability indices and proportional contribution to commercial vs subsistence landings (P = 0.005; R^2^ = 0.662, regression between vulnerability and the difference between commercial and subsistence landings, excluding rabbitfishes; Fig 6a).

## Discussion

The commercial reef fishery in Chuuk is larger than others across Micronesia, with over 260 mt of estimated annual landings (i.e.7-251 mt, Kosrae-Pohnpei) [30]. Yet, commercial extraction per reef area was 2.8 to 6.3 lower at Chuuk than other main islands in the Federated States of Micronesia due to the extensive coral-reef habitat in Chuuk (0.12 mt km^2^ year^-1^, compared to 0.32 and 0.75 for Kosrae and Pohnpei, respectively). Several indicators similarly suggested that a lower human fishing footprint existed when compared to other Pacific coral-reef fisheries as: i) no clear shifts in the spatial distribution of fishing effort or landings existed with seasonal trade wind patterns [30], ii) socioeconomic factors associated with demand were the main drivers of daily landings (i.e. air-freight connections to Guam), and iii) high proportional contributions from larger predators and secondary consumers existed [46–48]. While these attributes suggested diminished human fishing footprints in comparison to other fisheries, we also summarize that Chuuk’s spatial expansion to the remote barrier reef provided the highest and most consistent fishing success despite higher travel costs. In addition, skewed size structures and environmentally-constrained catch success were revealed for most target species. Therefore, the cumulative findings of this and previous studies reconciled that steady supplies of Chuuk reef fish to both local and export markets over the past decade may have masked potential growing fishing footprints and shifting populations of target stocks, thereby providing a false sense of sustainability.

The growing dominance of mid-sized species in landings while moving up in trophic position raises potential concerns for fishing down the food web. Theory suggests that linear relationships should exist between biomass in the food web and body size, and the select capture of large predators is a commonly reported trend for profit-driven harvesting [38,49–51]. Yet, assessing whether any particular species is naturally rare, rare because of differing catchability, or becoming rare due to fishing pressure remains challenging. While impacts of catchability in Chuuk remain unknown, high market demand for large-bodied species has recently been reported [23]. We therefore hypothesize the replacement of large-bodied species with smaller-bodied counterparts in accordance with trophic position may be more a consequence of fishing pressure and less a consequence of natural factors. In support, this same trend has been observed elsewhere in Kosrae, Micronesia [30,52], and on Guam [53]. In all these instances, mid-sized species that dominated landings of snappers and groupers had skewed size distributions indicating compensatory density dependence responses. A growing dominance would be expected for species with faster population growth rates, while less-resilient species would be expected to slowly diminish from landings. Unique to the present study, surgeonfish landings were dominated by two small-bodied species preferred by local consumers, *A*. *lineatus* and *N*. *lituratus*. These findings may have been driven more by preference and less by indiscriminate harvesting for large fish to maximize profit, as these species are typically sold for local consumption instead of export [54–56]

In addition, catch success was dependent upon environmental regimes. New moon phases and other environmental cycles are known to increase fishing success and landings, but the degree of dependence on favorable environmental regimes can grow as stocks become depleted [19,57,58]. Here, catch success for many mid-size predators, invertivores, and some herbivores was dependent upon seasonality and moon phases, albeit to a lesser degree than in other Micronesian jurisdictions [30]. For example, large catch contributions from the square tail grouper, *P*. *areolatus*, during the winter and spring were driven by large peaks in February and March (29% and 26% of overall spearfishing landings biomass, respectively), when they form large reproductive aggregations [59,60]. In contrast, herbivore landings were driven more by monthly moon phases. Increased night-time spearfishing catch-and-effort has generally been attributed to lower lunar illumination [59,61], but we found that proportional contributions also shifted as several species appeared most susceptible during specific moon phases (e.g. *N*. *unicornis* in new moon, and *Chlorurus microrhinos* in full moon). This could be related to reproductive cycles, and suggested the potential of identifying reproductive patterns from landings signals [62], or helping to guide sampling to determine reproductive cycles. In support, forktail rabbitfish, *Siganus argenteus*, had increased proportional contributions during the full moon phases, and supporting studies have confirmed the full moon corresponds with reproductive behavior [63]. Identifying if fishing success has increasingly become more dependent upon favorable fishing conditions will be possible as time series datasets become available. Regardless, targeting species when they are most vulnerable can provide short-term fisheries gains, but obviously threatens the sustainability of these populations [64].

In addition to environmentally-constrained catch success, many dominant mid-sized predators and invertivores were also found to have skewed size frequencies, indicative of compensatory density dependence and high fishing mortality compared to natural mortality. This classic population response may help maintain overall catch biomass in the short term, but truncated age- and size structures eventually lead to population instability because the fishery becomes more dependent on the success, or failure, of each recruiting cohort, and tracks environmental variability [36,45,65,66]. In contrast, most herbivores had normal size distributions suggesting minimal impacts to their size- and age-structure. Three exceptions were the browsing herbivores *N*. *unicornis*, *Kyphosus vaigiensis*, and *Kyphosus cinerascens*. These represent commonly harvested species across Micronesia that were previously found to have positive skewed size distributions as well as non-significant changes, or even increases, in proportional landings [5,30]. The current observations may therefore reflect a growing human fishing footprint into herbivorous fishes in Chuuk.

### Contrasting subsistence and commercial fishing

Shifts from subsistence to commercial fishing in recent decades have prompted a debate regarding the tradeoffs for coral-reef fisheries [3,11,67–69]. We contributed to this debate by contrasting commercial versus subsistence landings. Commercial fisheries in Chuuk preferentially targeted species with higher vulnerability to fishing, representing large-bodied fishes in higher trophic positions. In contrast, subsistence fishing targeted mostly small-bodied species with faster growth and turnover rates. The parsimonious explanation is that larger fish provide a greater profit for less effort, coupled with high consumer demand for large-bodied species in commercial and export markets. As an example, local demand for groupers in Chuuk is minimal due to strong cultural taboos. Yet, nearly 60 mt of groupers were exported to Guam in 2014, where demand for these species is high [23]. In contrast, subsistence landings were dominated by smaller resilient species, matching local nutritional preferences. This preference may have evolved from localized depletions of less resilient species in nearshore habitats targeted by subsistence fishers [28,70–72]. However, subsistence fishers do not usually have access to improved boats and gear to expand their fishing footprints due to high costs, leading to evolving nutritional preferences for smaller, faster growing species [73,74]. However, contrasting the impacts from subsistence and commercial fishing remains challenging and requires further study. Our findings point towards disproportional impacts of commercial fisheries due to the fishery expansions taking place and then fact that larger, long-lived species were targeted.

### Management

Multi-species, small-scale coral-reef fisheries are complex and dynamic. Yet, simplified management approaches can benefit from the present study. Species with significant declines in their size-structure, despite having large contributions to landings, represent candidates for size-based policies, such as maximum size limits, minimum size limits, or slot-based harvesting [30,75]. Such size-based policies are beneficial for species with high recruitment, and could preserve both spawning stock biomass and ecological functions [61]. Species without size responses that were preferentially caught during favorable moon phases or seasons may better respond to temporal/seasonal bans, catch quotas, or even gear restrictions [30,59]. These types of management measures aim to protect the fishable biomass of stocks, but do less to directly address the population size structure [76]. The geographical distribution of fishing effort also provided key guidance for simplified management. Commercial fisheries that focus almost exclusively on the remote barrier reefs may be best suited for management focused at the market level (i.e. minimum sale size), given the centralized location of fish markets, as spatial enforcement at the lagoon level would be extremely challenging and resource intensive. Alternatively, nearshore resources targeted primarily by subsistence fishers may be best suited for area-based management, as proximity and traditional resource ownership could maximize compliance and enforcement. Information from this study is being integrated into ongoing efforts to enhance fisheries management regimes while building common ground between Chuuk fishers, market owners, resources managers, and communities. Complementing existing area-based management with other forms of data-based, ecosystem-based fisheries policies would enhance livelihood sustainability as well as healthy ecosystems.

## Supporting information

S1 Fig Fishing success and profit at different reef habitats. Mean catch (a) and net income (b) for bottomfishing (black bars) and spearfishing (gray bars) within different reef habitats.
(EPS)

S2 Fig Fishing effort Vs landings. Number of fishing trips recorded and total landings from different barrier-reef sections (shapes) and seasons (colors).
(EPS)

S3 Fig Monthly fishing success. Mean monthly spearfishing landings (gray bars) and bottomfishing landings (black bars) with respect to the annual means (dashed lines).
(EPS)

S1 Tables **Table A**. Mean and overall landings data from different weekdays. **Table B**. Catch and profit data for different reef types and gear. **Table C**. Frequency of trips and overall landings data for different reef sections and seasons. **Table D**. Mean landings data across different winspeeds and distance to full-moon. **Table E**. Mean fisher catch-per-trip data across study months. **Table F**. Contribution to overall landings data of top families and species. **Table G**. Data for relationship between species maximum length (L_max_) and proportional contribution to family landings. **Table H**. Size frequency data for top 20 species. **Table I**. Daily contribution to landings data for top 20 species. **Table J**. Data for comparisons of vulnerability of landings from commercial vs subsistence fishing.
(XLSX)
